# Rapid, non-invasive, *in vivo* measurement of tissue mechanical properties using gravitational loading and a nonlinear virtual fields method

**DOI:** 10.1098/rsif.2023.0384

**Published:** 2023-10-11

**Authors:** S. L. Evans, B. E. Keenan, J. Hill, S. Zappala, N. Bennion, S. Avril

**Affiliations:** ^1^ School of Engineering, Cardiff University, The Parade, Cardiff CF24 3AA, UK; ^2^ School of Computer Science, Cardiff University, The Parade, Cardiff CF24 3AA, UK; ^3^ Mines Saint-Étienne, Univ Lyon, Univ Jean Monnet, INSERM, U 1059 Sainbiose, 42023 Saint-Étienne, France

**Keywords:** brain, virtual fields, mechanical properties

## Abstract

Measuring the mechanical properties of soft tissues *in vivo* is important in biomechanics and for diagnosis and staging of diseases, but challenging because it is difficult to control the boundary conditions. We present a novel, non-invasive method for measuring tissue properties using gravitational loading. MRI images of an organ in different positions are registered to measure tissue displacements due to gravitational forces in different positions. Considering equilibrium between stresses and gravity, we established a nonlinear virtual fields method to identify the tissue properties. The method was applied to the human brain as a proof of concept, using an Ogden model. Sensitivity analysis showed that the bulk modulus could be identified accurately while the shear modulus was identified with greater uncertainty; the strains were too small to identify the strain stiffening exponent. The measured properties agreed well with published *in vitro* data. The technique offers very promising perspectives, allowing the non-invasive measurement of otherwise inaccessible tissues and providing new information such as the bulk modulus under static loading, which has never previously been measured *in vivo*.

## 1. Introduction

Measuring the mechanical properties of soft tissues is important for analysis and simulation and has valuable diagnostic applications, notably in the liver but also in other tissues such as the breast, prostate and kidney [1,2], where stiffness measurements can provide an additional contrast and help to distinguish and stage disease. Since the properties of soft tissues are very variable between sites and subjects and in different environments it would be useful to measure them *in situ* in the body, but this is difficult since the boundary conditions cannot be well defined and controlled. Techniques such as shear wave elastography provide semi-quantitative measurements but only for very small strain perturbations, and they cannot easily be used in all tissues or measure all properties. Applying external loads is difficult for many tissues that are not easily accessible from outside the body. In this paper, we present a technique using gravitational loading, which offers a way of applying accurately known loads to otherwise inaccessible tissues, and we demonstrate the technique using a previously published dataset for the brain.

Since they are generally nonlinear and undergo large deformations, the mechanical behaviour of soft tissues is best represented by nonlinear constitutive models. The identification of the parameters in a constitutive model from experimental data is often difficult [3,4]. The parameters cannot often be calculated directly, as they can in conventional tests on engineering materials, and an iterative approach is usually needed to estimate their values. The most common approach has been the so-called ‘inverse FE’ or ‘FE model updating’ method in which a finite-element model of the experiment is constructed and the material parameters are iteratively optimized to try to match the experiment [3,4]. Although widely used, this method has two significant disadvantages: because hundreds or thousands of runs of a complex, nonlinear model are needed, it can be very time-consuming, and there is no immediate way of knowing whether the identified parameters are the best possible fit to the experimental data, or what range of other values might also be possible. In the worst case, where certain parameters have little effect on the quality of fit between the experiment and the model, it may be impossible to identify them. For example, Masson *et al*. [5] attempted to identify parameters of a complex four-fibre model from *in vivo* data but found that many parameters had little effect on the response.

These limitations have been addressed in various ways, for example by using stochastic optimization in which multiple models can be run in parallel [6,7]. Multiple optimization runs from different starting points can be used to indicate whether global convergence has been reached, and mapping the response surface for a range of possible parameter values can show the uncertainty and uniqueness of the parameter values. Often two parameters have a similar effect, so that increasing one and decreasing the other gives a similar error, and then it is difficult to know which is correct without further experiments. Sensitivity analysis can help to characterize and explore these problems [3].

A more efficient approach is to use a small number of model runs to explore the possible parameter space and produce an emulator that will predict the model output and can then be used to test a very wide range of parameter values and explore their uncertainty and uniqueness in detail [8,9]. Using Bayesian statistics, it is also possible to quantify the uncertainty in the numerical simulation and build in some *a priori* knowledge of the smoothness of the response surface. However, this approach still requires tens or hundreds of runs of a nonlinear model.

The virtual fields method [10,11] offers the possibility of calculating material properties directly from strain measurements for linear elastic materials, or, for nonlinear materials, of calculating an error measure directly from strain data without solving an FE model. This has the potential to be several orders of magnitude faster than the inverse FE approach, allowing rapid parameter identification and uncertainty evaluation. The method is based on the principle of virtual work, which states that if a hypothetical virtual displacement is applied to a system, the resulting virtual work done by the internal and external forces must sum to zero. By choosing appropriate virtual displacement fields, we can isolate individual materials or components and remove inconvenient unknown boundary conditions. The method thus allows us to sum the stresses in a material or region and test whether they are in equilibrium with the external forces. If we calculate the stresses from a measured strain field using a particular constitutive model and set of parameters, we can test whether the internal forces it predicts are consistent with the known external forces, and any difference gives us a measure of the error in the model and its parameters.

Applying the virtual fields method to nonlinear, large-displacement problems is mathematically complex and requires care to avoid errors in calculating rates or using incorrect frames of reference. Rossi *et al*. [12] and Jones *et al*. [13] used virtual fields techniques to identify material parameters during plastic deformation of stainless steel, using two-dimensional strain data measured by digital image correlation. A systematic approach to large deformation parameter identification using virtual fields was presented by Mei *et al*. [14], in which they calculated the sensitivities of the stresses to the material parameters and hence produced a system of equations that could be solved to find the parameters. This provided a framework for the choice of virtual fields, multiple fields being needed in general where there are multiple material parameters to identify. For simple cases, it is possible to use arbitrary fields such as a linearly increasing virtual displacement in one direction, but for more complex cases it is better to generate the fields using linear finite-element models.

The aim of this study was to use a novel large deformation formulation of the virtual fields method to identify the material properties of the brain *in vivo*, using displacement data from a previous study [15] in which we magnetic resonance imaging (MRI) scanned subjects lying prone and supine and used image registration to calculate the displacement field. We also used a finite-element model to generate simulated displacement data to optimize the image registration process and test the accuracy of the method.

## 2. Methods

### 2.1. Theoretical framework

We use the weak form of solid mechanics [16], similar to the formulation used by Rossi *et al*. [12] but with the addition of body forces,

2.1 $$\int_{V}\sigma :D^{*}dV=\int_{V}(b-a)\cdot v^{*}\rho dV+\int_{S}(\sigma \cdot n_{t})\cdot v^{*}dS,$$

where **σ** is the Cauchy stress tensor, **a** is an acceleration force vector, **b** is a body force vector, **n**_t_ is a surface traction acting on a solid of volume *V*, surface *S* and density *ρ*; v* is an arbitrary test function, considered here to be a virtual velocity field, and D* is the virtual rate of deformation tensor defined by

2.2 $$D^{*}=\frac{1}{2}(\nabla v^{*}+\nabla^{T}v^{*})$$

The volume, surface area and density are in the current configuration, not the undeformed configuration, and the gradient of the virtual velocity field is with respect to the current position, not the original position. It is possible to formulate the same principle in the reference configuration, but that would require changes to the stresses and surface tractions too [12,17].

In the virtual fields method, the test function v* is usually conceived as a virtual displacement and an equation similar to equation (2.1) then follows from the principal of virtual work, in which the virtual work done by a virtual deformation should be zero. For linear problems, this virtual work is easy to calculate by multiplying the actual stress by the virtual strain (and the actual forces by the virtual displacements), but for nonlinear problems this integration becomes more complicated than a simple multiplication and it would be necessary either to assume that the virtual displacement is infinitesimal compared with the actual displacement or to use numerical integration over multiple steps to find the virtual work. Here we describe the test function v* instead as a virtual velocity, which avoids these conceptual problems. It should be noted though that these problems arise only from the concept of the test function as a virtual displacement and in fact any arbitrary function that can be differentiated in equation (2.2) can be used. The idea of a test function is perhaps clearer, and it avoids the confusion between the virtual and real displacement fields that often arises with the conventional virtual fields terminology. Similarly, in the conventional virtual fields terminology there are additional restrictions on the test function to make it *kinematically admissible*, but these are not inherent to the weak form in equation (2.1) and we prefer to describe them below as convenient ways to choose the function to suit the available data, rather than inherent restrictions.

The left-hand side of equation (2.1) describes the *internal virtual power* due to the stresses within the material and the right-hand side describes the *external virtual power* due to external loading. We aim to choose virtual fields that will allow us to calculate the internal virtual power by calculating the stress from the measured deformation, using a suitable constitutive model, and adjust the material parameters so that it matches the external virtual power found from known external forces.

The virtual velocity field (or test function) v* is arbitrary, and in principle any differentiable function of the position should satisfy equation (2.1), but a careful choice can allow us to isolate individual materials, remove unknown boundary conditions and maximize the identifiability of the parameters. By choosing a velocity field which is zero except in a single material, component or region of interest, we can isolate the contribution of that region and identify its properties independently of any other materials that may be present. If there is sufficient resolution in the measurements, this method can be used to map out the spatial variation of properties. Similarly, if there are unknown surface tractions, we can choose a velocity field that is zero in the region where the unknown traction is applied, so that the third term in equation (2.1) disappears. We cannot identify material properties from displacements alone, however; we need a known force somewhere in the system, so that the external virtual work is non-zero. There are two convenient techniques we can use to achieve this. If we know the total force applied over a region of the surface but not the distribution of that force, we can choose a velocity field which is constant over the region and calculate the resulting external virtual power as the product of the force and the virtual velocity without the need to integrate over the surface of the region. Secondly, if there is a known body force such as gravity, we can choose a virtual field which is zero at all of the unknown boundaries, so that the third term of equation (2.1) disappears, and use only the first and second terms.

In order to solve equation (2.1) numerically, we need to evaluate the internal and external virtual power at a series of integration points and sum over the volume of interest. These integration points could be defined in various ways, but it is convenient to define them by a finite-element mesh representing the region of interest. This finite element model can be used to calculate suitable virtual fields using any convenient linear material properties, as described below. The measured displacement field can be interpolated to find the displacements at the nodal coordinates, and conventional finite-element calculations can then be used to find the resulting stresses and integrate them over the volume of the elements to find the internal and external virtual power [18].

### 2.2. Magnetic resonance imaging and image analysis

The measurement of the movement of the brain has been described in detail in a previous paper [15]. Briefly, three male subjects aged 30–60 were imaged. Each subject was positioned prone in either a Siemens 7T Magnetom or a Siemens 3T Prisma MRI scanner and after resting in that position for 20 min to allow the brain to reach equilibrium, they were scanned using a T1-weighted MPRAGE sequence with either 0.8 × 0.75 × 0.75 mm resolution (7T scanner) or 1 mm isotropic resolution (3T scanner). They then turned over into a supine position and were scanned again, 10 min after the first scan. In a pilot study, subjects were repeatedly scanned at shorter intervals after turning over, using a faster two-dimensional sequence, in order to verify that the brain had reached equilibrium after 10 min, the time of the second scan.

Extensive distortion correction was applied using GradUnwarp (github.com/Washington-University/gradunwarp) [19] as well as proprietary algorithms in the Siemens software to minimize image distortion, which was a particular problem with the 7T scanner. A semi-automated segmentation process was carried out to extract the skull and brain masks, using the brain extraction tool (BET) command of the FSL software library [20]; the segmentations were amended manually where necessary using Seg3D (Scientific Computing and Imaging Institute).

The prone and supine images were then registered using the symmetric image normalization (SyN) method [21], starting with an affine registration of the skull only to align the head, followed by an elastic registration of the brain and surrounding soft tissues. The registration algorithm was optimized using simulated images generated with a displacement field from the finite-element model, in order to find the registration parameters that most closely recaptured the known displacement field. This allowed very accurate measurement with subpixel resolution. The displacement data were then mapped to the MNI152 average brain [22] and averaged for all the subjects to provide a single experimental displacement field.

### 2.3. Finite-element model

**Figure 1.**
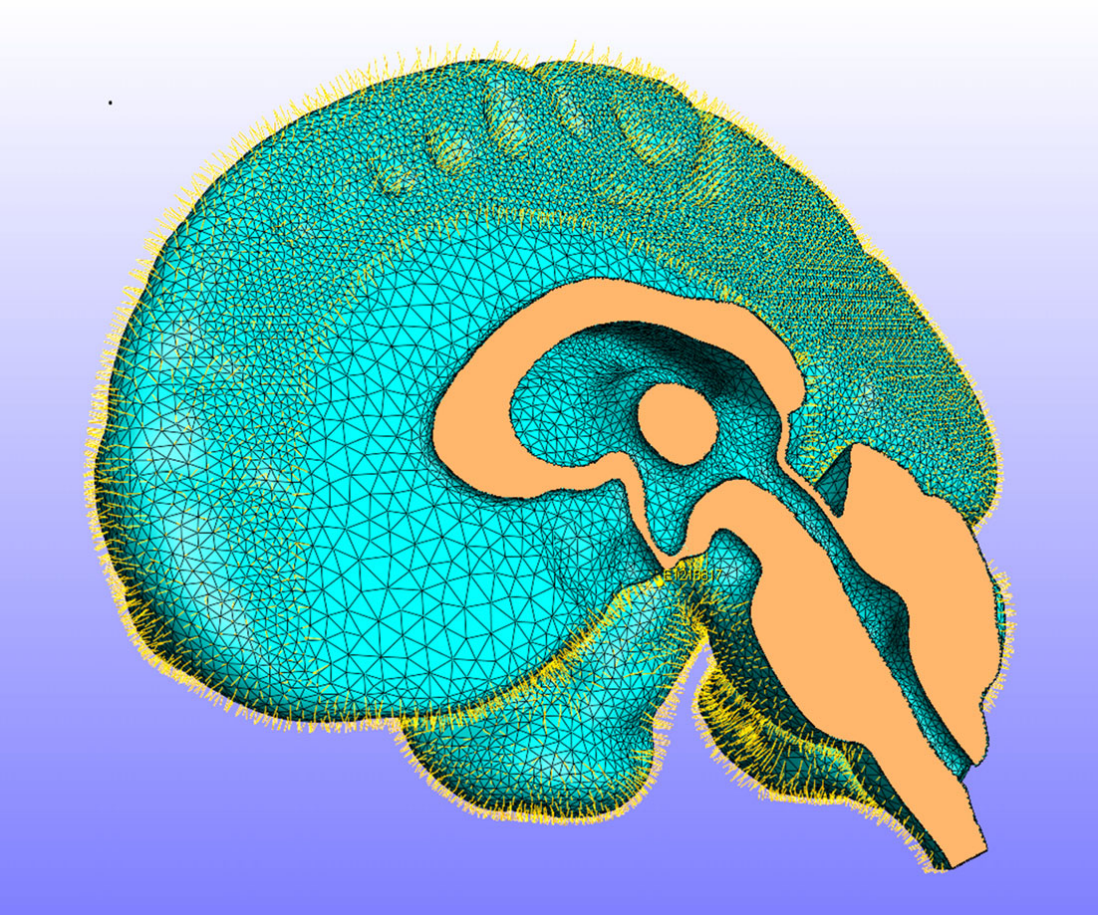
The finite-element model of the brain: the right side of the model showing the ventricles and the fluid-filled subarachnoid space with spring elements representing the arachnoid trabeculae. The dura mater and CSF have been hidden for clarity.

It is convenient to calculate the virtual fields using a finite-element model, which also provides a framework for calculating the internal and external virtual power. For this study, we used a more elaborate finite-element model of the brain, dura and cerebro-spinal fluid (CSF) [23] which was used in its full form to generate simulated displacement data in order to verify the virtual fields algorithm. This model was based on the MNI 152 standard space [22]. Images were segmented using Simpleware ScanIP (Synopsys, Mountain View, USA) and manually optimized. The final model consisted of three main volumes: the brain, the CSF-filled space and the dural septa. The model was meshed in ScanIP using four noded tetrahedral elements, with 432 059 elements for the brain, 367 752 elements for the CSF and 376 309 for the dural septa. The model was then imported into FEBio [24, www.febio.org] and modified using Matlab (Mathworks, Natick, MA, USA) to add an additional layer of shell elements to the surface of the brain to represent the pia mater and spring elements bridging the dural space to represent the arachnoid trabeculae, as shown in figure 1. The brain was modelled as an Ogden material, the pia mater as a neo-Hookean material and the dura mater as a rigid body as it was assumed to be fixed to the skull. The CSF, including in the dural septa, was modelled as a Newtonian fluid.

It is important to note that the full model was used only to generate test data to verify the implementation of the virtual fields algorithm. For the latter, only the mesh of the brain itself was needed, firstly to generate the virtual fields and secondly for the calculation and integration of the virtual work. For the virtual fields, we need a displacement field which is zero at the boundaries of the brain (where there are unknown tractions), so all nodes on the boundary of the brain and in the other tissues were fixed and a body load was applied to the brain to generate a displacement field. The body load was smaller than the actual gravitational load (0.001 m s^2^), in order to ensure that the deformations were small. The model was then run again with different values of each of the material parameters in turn and the first virtual field was subtracted from each of these fields, in order to produce three fields that best represented the effects of each of the material parameters, which were then used as the virtual velocity fields.

### 2.4. Numerical implementation

The virtual velocity fields were chosen to be equal to the displacement **u** in the finite-element models that were used to generate them. The gradient of the virtual velocity was calculated from the displacement **u** and the resulting deformation gradient **F** using the chain rule as follows:

2.3 $$\nabla u=\frac{\partial u}{\partial x}=\;\frac{\partial u}{\partial X}\frac{\partial X}{\partial x}=(F-I)F^{-1},$$

where **X** is the original position, **x** is the current position and **I** is the identity tensor.

The internal and external virtual power were discretized using a single integration point at the centre of each element, which is sufficient for four node tetrahedral elements, which have constant strain throughout their volume. The internal virtual power was calculated as

2.4 $$P_{\mathrm{int}}\equiv \int_{V}\sigma :D^{*}dV\approx \overset{N}{\underset{i=1}{\sum }}\sigma :D^{*}\cdot JV_{i}^{0},$$

where *N* is the number of elements, *V^0^_i_* is the undeformed volume of element *i* and *J* = det **F** is the ratio of the deformed to the undeformed volume. For the brain under gravitational loading, there are no other acceleration forces (**a**) and surface tractions were eliminated by choosing a virtual field that was zero at the surfaces, so the external virtual power was calculated as

2.5 $$P_{\mathrm{ext}}\equiv \int_{V}(b-a)\cdot v^{*}\rho dV\approx \overset{N}{\underset{i=1}{\sum }}b\cdot v^{*}\cdot \left(\frac{\rho }{J}\right)JV_{i}^{0}=\overset{N}{\underset{i=1}{\sum }}b\cdot v^{*}\cdot \rho V_{i}^{0},$$

in which *ρ* is the original density of the material and hence *ρ/J* is its density in the deformed configuration.

The brain was modelled as a compressible Ogden material [25] with the following strain energy function, also used in the virtual power calculation:

2.6 $$\psi =\sum \frac{2\mu }{\alpha^{2}}({\tilde{\lambda }}_{1}^{\alpha }+{\tilde{\lambda }}_{2}^{\alpha }+{\tilde{\lambda }}_{3}^{\alpha }-3)+U(J),$$

in which *μ* is the small strain shear modulus, *α* is a material parameter describing stiffening at large strains, ${\tilde{\lambda }}_{i}$ are the deviatoric principal stretches and *U*(*J*) is a volumetric term such that the volumetric part of the Cauchy stress is given by

2.7 $$\sigma_{V}=K\frac{\ln(J)}{J}I,$$

in which *K* is the bulk modulus of the material.

These calculations were implemented in a Matlab script (Mathworks, Natick, MA, USA) which read in the element connectivity and node coordinates from the FE model, the experimental displacements at each node, and the virtual fields. The deformation gradients and element volumes were calculated using conventional finite-element mathematics [18] and used to calculate the stresses and hence the internal and external virtual power for different combinations of material parameters.

### 2.5. Parameter identification

Having calculated the internal and external virtual power *P*_int_ and *P*_ext_ for each virtual field, the virtual power error for each virtual field *j* was defined as the difference between them, normalized by the external virtual power, which is independent of the material properties,

2.8 $$P_{\mathrm{err}}^{j}=\frac{P_{\mathrm{int}}-P_{\mathrm{ext}}}{P_{\mathrm{ext}}}.$$

The total virtual power error was then defined as the Pythagorean sum of these errors for the individual fields,

2.9 $$P_{\mathrm{err}}^{\mathrm{total}}=\sqrt{P{{\;}_{\mathrm{err}}^{1}}^{2}+P{{\;}_{\mathrm{err}}^{2}}^{2}+P{{\;}_{\mathrm{err}}^{3}}^{2}}.$$

This error was then minimized using two different algorithms, Levenberg-Marquardt (Matlab *lsqnonlin*) and Nelder–Mead simplex optimization (Matlab *fminsearch*), and each was repeated from multiple startpoints. To assess global convergence and uncertainty, 2800 random parameter sets over a feasible parameter space were also tested.

### 2.6. Effects of measurement errors

In order to test the effect of noise in the displacement data, simulated data from the FE model were used with the addition of varying amounts of noise, generated by adding normally distributed random values to each displacement component with a mean of zero and a standard deviation corresponding to the RMS noise level. These synthetic displacement data were processed as before and the resulting errors in the internal virtual power were evaluated. This process was repeated twice for three different noise levels (0.25, 0.5 and 1 μm RMS). A further test was carried out by using a synthetic MRI image generated by warping an image of one of the subjects using a displacement field generated from the FE model; this synthetic image was then used in the registration process to generate a displacement field with typical registration errors. The results from this synthetic image were compared with the known displacement field from the FE model to simulate the actual errors likely to arise in the registration process.

## 3. Results

**Figure 2.**
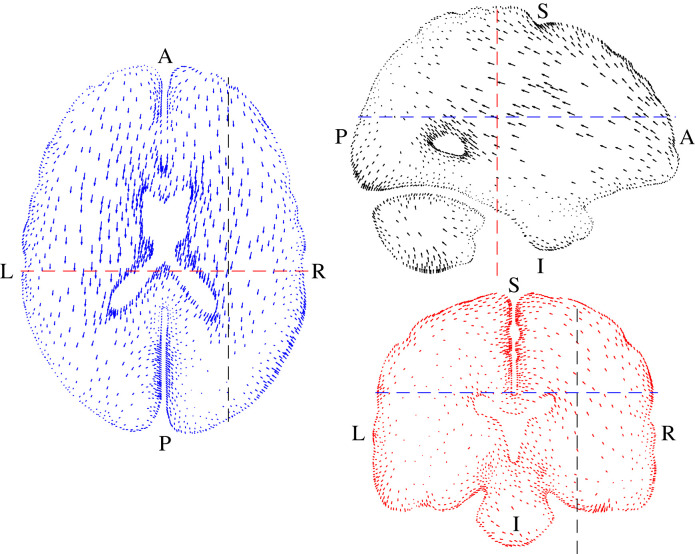
Displacement data from the MRI measurements [15]. The vectors show the displacements, scaled by a factor of 5 for clarity. Letters show anatomical directions: A = anterior, P = posterior, S = superior, I = inferior, L = left, R = right; transverse section (left), sagittal section (top right) and frontal section (lower right); the position of each section is indicated by the dashed lines in the other views which match the colours of the vectors in each view.

Figure 2 shows the average displacement data. The movement of the brain was predominantly in the anterior–posterior direction but there were also some rotations and lateral movements. The movement occurred primarily by deformation of the brain itself with only small displacements near the outer surface where the brain is tied to the skull by the pia-arachnoid complex. The maximum displacement was 1.15 mm in the left side of the cerebellum.

**Figure 3.**
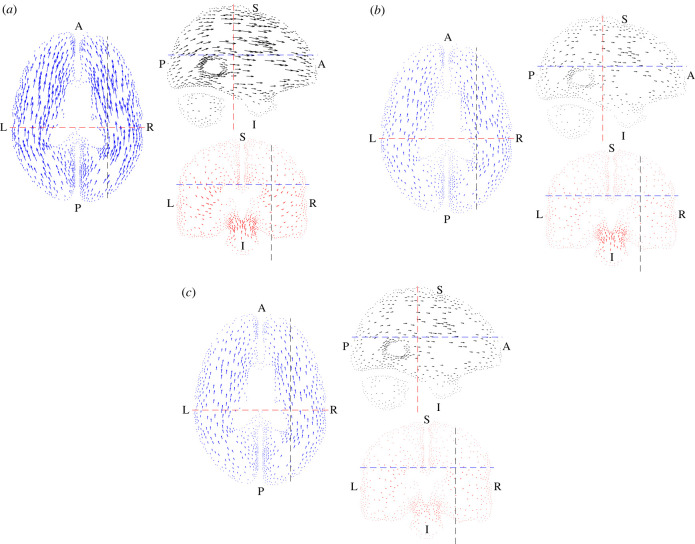
(*a*) The first virtual field optimized to identify *μ*, plotted in the same way as figure 2 but with the vectors scaled by 10^5^. (*b*). The second virtual field optimized to identify *α*, plotted in the same way as figure 2 but with the vectors scaled by 10^5^. (*c*) The third virtual field optimized to identify *K*, plotted in the same way as figure 2 but with the vectors scaled by 10^5^.

Figure 3 shows the three test functions v* that were used to identify the material parameters. In each case, the function was zero at the outer surfaces in order to remove the effects of the unknown boundary conditions where the brain is attached to the skull. We evaluated the internal and external virtual power *P*_int_ and *P*_ext_ and defined the virtual power error as the difference between them. The total virtual power error was then defined as the Pythagorean sum of these errors for the three fields.

**Figure 4.**
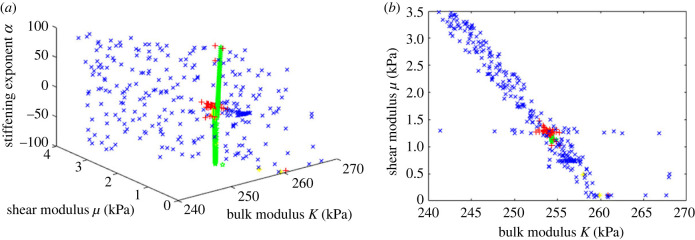
Error function $P_{\mathrm{err}}^{\mathrm{total}}$ plotted as a function of the material parameters from multiple optimization runs using two different optimization algorithms: green stars show parameter sets with an error less than 1 × 10^−8^, yellow asterisks 1 × 10^−8^ $<\;P_{\mathrm{err}}^{\mathrm{total}}$ < 1 × 10^−6^, red + signs 1 × 10^−6^ $<\;P_{\mathrm{err}}^{\mathrm{total}}$ < 1 × 10^−4^ and blue cross symbols 1 × 10^−4^ $<\;P_{\mathrm{err}}^{\mathrm{total}}$ < 0.01. (*a*) the parameter space shown in three dimensions; (*b*) projection on to the *μ*–*K* plane. Extremely small errors were possible for any value of *α* but the other parameters were identified consistently and precisely.

This error was then minimized using two different algorithms, Levenberg–Marquardt (Matlab *lsqnonlin*) and Nelder–Mead simplex optimization (Matlab *fminsearch*). Figure 4 shows the results of the optimization process, using both algorithms from multiple start points. Both algorithms produced a range of possible values with very small errors. *K* and *μ* were identified precisely, with values around 254 kPa and 1.25 kPa, respectively. Parameter sets with negligible error were possible for any value of *α* from −100 to +100, and so it was not possible to identify this parameter.

**Figure 5.**
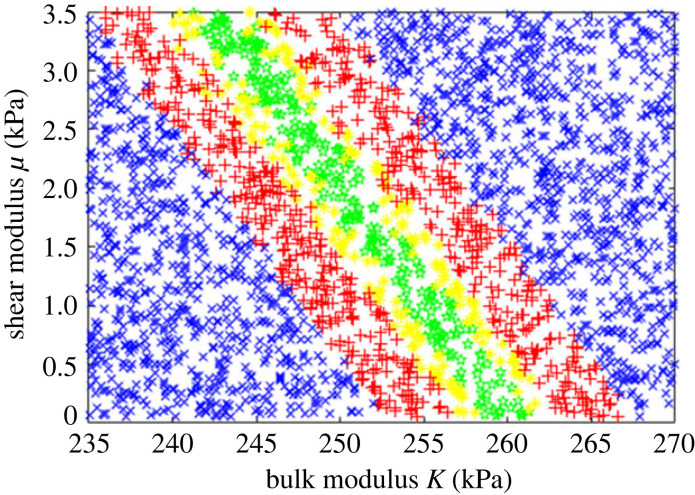
Errors for 2800 random parameter sets. Green stars show points with errors less than 1%, yellow asterisks 1% $<\;P_{\mathrm{err}}^{\mathrm{total}}$ < 2%, red plus signs 2% $<\;P_{\mathrm{err}}^{\mathrm{total}}$ < 5%, blue cross signs $P_{\mathrm{err}}^{\mathrm{total}}$ > 5%. Note the axis scales: *K* was identified with very little uncertainty, but a much wider range of values for *μ* result in only small errors.

Figure 5 shows the errors for 2800 random parameter sets as a function of the material parameters, showing the uncertainty in the identification process. Allowing even substantial errors resulted in very little change in *K* (note the limited scale), but a much wider range of values for *μ* was possible.

**Figure 6.**
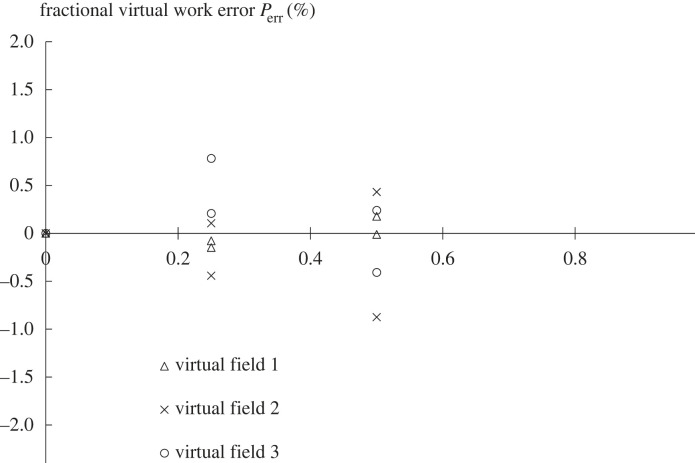
The effect of adding synthetic noise to the displacement data. The graph shows the values of the error function *P*_err_ plotted against the RMS noise level for each of the three virtual fields; this was repeated twice so that there are two sets of points. It is evident that there was no systematic bias in the error function $P_{\mathrm{err}}^{\mathrm{total}}$ as a result of adding noise and that the three virtual fields were affected independently and differently.

Figure 6 shows the change in the virtual field error for each field when adding increasing amounts of synthetic random noise. There was no systematic bias due to noise and each field was affected differently and independently.

The effect of realistic errors in the image correlation process was assessed by registering synthetic images with a known, realistic displacement field, generated from the FE model. This produced an error in the virtual power of less than 5%, suggesting that the accuracy of the stiffness measurement should be of this order.

The stresses with the optimized parameters were very low and approximately normally distributed. Ninety-five per cent of elements had shear stresses smaller than 30 Pa; the maximum was 160 Pa. Larger triaxial stresses were present, with 95% of elements between −10.9 kPa and 10.1 kPa, compared with an expected hydrostatic pressure of approximately 2 kPa.

## 4. Discussion

The virtual fields method (VFM), or family of methods, have many advantages, but they have not been widely used due to their perceived mathematical complexity and the need for full-field displacement data. Previous formulations of the VFM have mostly been based on virtual work, which is straightforward to calculate for linear elasticity but more problematic for nonlinear systems with large displacements, as conceptually it would require either numerical integration over multiple steps or an assumption that the virtual deformation is infinitesimal [14]. The formulation presented here using virtual power is simpler and easier to implement for large displacements. The identity with the weak form of continuum mechanics is also valuable and gives confidence in the robustness of the approach. In the weak form, the virtual velocity field is usually referred to as a test function, which is perhaps less confusing and makes it clear that this is an arbitrary function used to test for equilibrium and not a real velocity or displacement field. This also confirms that any appropriate test function could be used and there is no need for it to be infinitesimal compared with the actual displacements or to carry out an additional numerical integration; these requirements appear only from the concept of the test function as a virtual displacement, and they are not mathematically necessary for a correct solution.

The virtual power must be numerically integrated over the volume of interest, and this could be done in many different ways. Using a finite-element mesh has several advantages: it is easy to construct, optimize and evaluate using well-established mathematics and software, it allows the virtual fields to be generated easily, and it can be used to generate simulated data to test the algorithm, the sensitivity of the parameters and the effects of noise. It is also an efficient way of representing the deformation field without requiring a very large number of integration points.

This virtual fields method is much faster than the conventional inverse finite-element approach. The computation required to evaluate one parameter set is equivalent to a part of a single iteration for a nonlinear finite-element model, where the solution requires thousands of iterations. The deformation gradients, element volumes and external virtual power need only be calculated once and so the calculation of the stress and internal virtual power is very fast, allowing efficient exploration of many parameter values. This makes it possible to explore thoroughly the sensitivity of the different material parameters and the uncertainty in their identification, which is often impractical with an inverse FE approach. The calculation of the stress and internal virtual power is done on an element-by-element basis, so it can be parallelized efficiently. We used the same finite-element model with a Bayesian emulator approach to estimate the material parameters using displacement data from a larger group of subjects [23] and this required 120 runs taking 12–24 h each on a supercomputer using 12 2.4 GHz cores, plus subsequent processing to generate the emulator and evaluate numerous possible parameter sets. By contrast, the present method required only a few hours of computing time using uncompiled Matlab, which could be reduced to a few minutes with more efficient code. An inverse finite-element approach would have required thousands of model runs and was prohibitively slow.

The displacements due to gravitational loading are somewhat larger than those due to the cardiac cycle (maximum 1.15 mm, in the cerebellum), whereas cardiac movement is typically less than 0.5 mm [26]. Magnetic resonance elastography typically applies much smaller displacements of around 5 µm [27,28] though some studies have used much larger displacements up to ±60 µm [29]. Another method that has been used is mild impact loading [30] which produces similar strains to the present method, although the displacement is unclear. All of these methods produce only small displacements and small strains and hence none of them allow measurement of the nonlinear behaviour of the brain at larger strains; it is difficult to see how this could be achieved *in vivo* in human subjects. The displacements are smaller in all cases than the MRI voxel resolution and noise is a significant issue for all these techniques.

The effect of noise in the displacement measurements was assessed by using simulated data generated by a finite-element model to which noise was added. Using random, normally distributed noise added independently to each displacement component caused an increasing error, as shown in figure 6, but did not lead to a systematic bias in the results. Each virtual field was affected differently and independently, so that averaging the results from multiple virtual fields could reduce the effect of noise. Since the elements were small, adding even small amounts of random noise generated significant strains and hence significant errors. A real error field is not random but arises mostly from the image registration process which imposes continuity and some smoothing on the displacement field. Testing using an actual error field generated by registering an artificial image resulted in a similarly small error (5.6%), even though the RMS error was many times larger. This is less than the difference between the three virtual fields andis acceptable for this type of measurement.

The optimization process using both algorithms and multiple start points converged in every case to a shear modulus of around 1.2 kPa and a bulk modulus of 254 kPa. However, solutions were found with negligible errors (less than 10^−6^) over the whole range of values of *α* from −100 to +100. For positive values of *α*, there was a weak coupling with the shear modulus while for negative values the shear modulus was constant at around 1.25 kPa. Since *α* is generally believed to be negative, this latter value is most likely to be correct. Budday *et al*. [31] reviewed numerous experimental measurements in the range 0.1–3 kPa, with the majority in the range 0.5–1.0 kPa. Since different test methods give different results and there are significant regional variations, it is difficult to be more precise, but clearly the present results match well with experimental data for *in vitro* brain tissue. Elastography measurements by Hiscox *et al*. [27] gave a shear modulus in the range 2.21–3.76 kPa, while Sack *et al*. [32] measured shear moduli from 2.17 to 4.50 kPa. These are higher than our measurements, but this is to be expected as elastography uses a much higher frequency of loading, which will increase the apparent stiffness of the tissue. The measured shear and bulk moduli correspond to a Poisson's ratio of 0.4977, which is consistent with literature data and typical assumptions about near incompressibility, and to a Young's modulus of 3744 Pa.

The gravitational deformation of the brain was too small to allow full characterization of the material properties. The Ogden exponent *α* describes stiffening of the tissue at large strains and could not therefore be determined from an experiment in which only very small strains were applied. A very wide range of values are reported in the literature (from +34.8 to −73.5 in Budday *et al*. [33], for example), suggesting that in general *α* is variable and difficult to measure accurately. The ability to recognize where parameters cannot be identified accurately is an important advantage of this method over the usual inverse finite-element approach.

In order to explore the uncertainty in the parameter identification process, it is informative to see how the range of possible parameter values increases if small errors are allowed in the virtual power. Figure 5 shows the error distribution for 2800 random parameter sets; while the bulk modulus can still be identified precisely, allowing small errors greatly increases the uncertainty in the shear modulus. Since the brain is constrained by the skull, it is difficult for it to move unless regions in compression reduce in volume and those in tension expand; movement of the brain is very dependent on movement of CSF within and around it. Volumetric stiffness is therefore the most important factor determining the amount of deformation and hence it could be identified most accurately. Shear stiffness and strain stiffening have much less effect and so could not be identified so precisely. Significant volumetric strains were found; in preliminary experiments it was shown that the brain reached equilibrium within a few minutes and so this represents the drained configuration after poroelastic fluid flow through the tissue has taken place. This is different from impact experiments or other situations where rapid loading occurs.

Budday *et al*. [34] and Voyiadjis *et al.* [35] demonstrated that the compressibility of brain tissue is different in tension, compression and shear and a more complex material model is required to represent this, so perhaps it is not surprising that it was difficult to identify unique values for the shear stiffness and bulk modulus in this study, where different parts of the brain experienced different combinations of tension, compression and shear. A more complex model with multiple terms could fit the measured behaviour better, although there may be insufficient information in the measurements to allow the identification of several more material parameters.

In our previous study using the same finite-element model with data from a larger group of subjects [23], we found average values of *μ* = 670 Pa, *K* = 148 kPa and *α* =−19, which compare well with the present results, although *K* was lower with much more uncertainty. It was possible to estimate all three parameters, but the present method quantified *K* much more precisely and enabled a systematic error analysis which revealed uncertainty in *μ* and *α*.

One limitation of this approach, which applies also to other methods such as the inverse finite-element approach, is that errors in the finite-element model such as insufficient mesh refinement will bias the results. This technique provides a convenient way to test for such errors, by calculating the internal and external virtual power for simulated displacements, which should match accurately. For the simplified models used to create the virtual fields, each can be tested using its own output as both the displacement field and the virtual field. This is a useful technique that may have other applications in assessing the accuracy of FE simulations.

Another important limitation is that full-field displacement data is needed. In the past, this has typically limited the use of virtual fields methods to structures such as thin plates where the full displacement field can be inferred from measurements at the surface, but increasingly it is possible to measure full three-dimensional displacement fields using techniques such as MRI, and there is an increasing interest in measuring mechanical properties of tissues from such images. It is to be hoped, therefore, that the technique may find useful applications.

The virtual power formulation includes an acceleration term which could be used to include dynamic effects such as wave propagation [36]. Since shear wave propagation depends on shear stiffness, a combination of the present technique (which is more sensitive to bulk modulus) with shear wave elastography might provide more complete parameter identification.

Using gravitational loading also has some valuable advantages. The density of most tissues is accurately known and so the load can be precisely quantified. The boundary conditions are also well defined and well understood. Gravity acts equally on inaccessible tissues deep within the body, such as the brain, loading them precisely and non-invasively. This is a valuable tool that has great potential for measuring the properties of internal organs such as the liver, where stiffness measurements have considerable diagnostic value in quantifying fibrosis and other diseases. In the brain, which is supported on all sides by the skull and CSF, the gravitational deformation is small and difficult to measure accurately and only a limited amount of material property information could be extracted assuming the tissue properties were constant throughout the whole organ. For tissues such as the breast, which undergo much larger gravitational deformations, it should be possible to obtain much more detailed information and potentially to map out tissue properties and hence delineate and assess suspected tumours.

## Data Availability

Data, code and the finite-element model are available from: https://osf.io/b5e6s/. The code consists of two Matlab files, VFM_22.mlx which inputs and processes the data, and optvf.mlx which optimizes the material properties. A finite-element model Dura_Prone.feb, a set of MRI displacement data avgT_uw_mesh.mat and three virtual fields are also provided.
